# *In Vitro* and *In Vivo* Biochemical Evaluations of the Methanolic Leaf Extract of *Garcinia kola*

**DOI:** 10.1155/2014/391692

**Published:** 2014-10-30

**Authors:** Jelili A. Badmus, Olaniyi T. Adedosu, Emmanuel G. Adeleke, Kehinde H. Akinboro, Bayonle I. Odeyemi, Bolanle I. Ayoola, Donavon C. Hiss

**Affiliations:** ^1^Department of Biochemistry, Ladoke Akintola University of Technology, PMB 4000, Ogbomosho 210211, Nigeria; ^2^Department of Medical Biosciences, University of the Western Cape, Private Bag X17, Bellville 7535, South Africa

## Abstract

*Garcinia kola *Heckel (*Guttiferae*) leaves have received limited scientific attention despite their traditionally acclaimed medicinal properties. The scavenging ability of the methanolic leaf extract (MLE) of* G. kola *was assayed for hydroxyl radical (OH^•^), superoxide anion (O_2_^−^), 1,1-diphenyl-2-picrylhydrazyl (DPPH), azinobis-3-ethyl-benzothiazoline-6-sulfonic acid (ABTS^•+^), and lipid peroxidation (LP) activity in egg yolk, rat liver, and brain homogenates. Total phenolic and flavonoid contents of the extract were also evaluated. Group I animals were given oral doses of water, whereas Group II and Group III animals received 100 and 200 mg/kg body weight (bw) MLE, respectively, for 14 days. Plasma glucose, magnesium, γ-glutamyltransferase (GGT/γGT), alanine aminotransferase (ALT), aspartate aminotransferase (AST), creatinine, and urea were evaluated. Hepatic reduced glutathione (GSH), glutathione peroxidase (GPx), superoxide dismutase (SOD), LP, and liver histopathological appearance were also assessed. The extract scavenged OH^•^, O_2_^−^, DPPH, and ABTS^•+^ and inhibited LP in egg yolk, rat liver, and brain homogenates. Furthermore, oral administration of the extract showed no adverse effects on hepatic and renal function tests. Increased hepatic GSH and nonsignificant changes in LP, GPx and SOD activities, and liver histology were observed. These results suggest that* G. kola leaves* have antioxidant activities which may have application in traditional medicine.

## 1. Introduction

Medicinal plants have been used as folklore remedies over the years to treat and manage human ailments. A large body of scientific evidence has been collated to show their immense potential in various traditional cultures [1]. They contain a large variety of chemical substances that possess important therapeutic properties used in the treatment of innumerable diseases [2]. These chemical substances generally occur as secondary plant metabolites, including saponins, tannins, essential oils, flavonoids, and alkaloids [3]. The medicinal potential of natural plant products has led to growing research into the discovery of novel drugs as alternatives to synthetic ones whose beneficial effects are often eclipsed by severe toxicities and adverse events.

**Figure 1:**
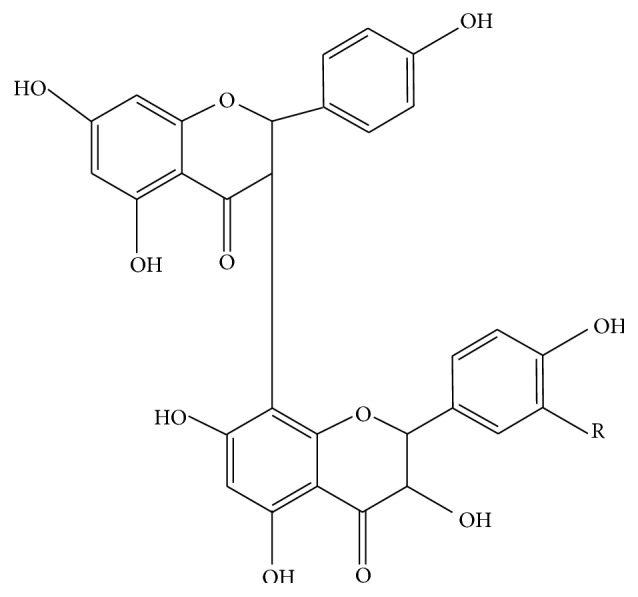
The structure of the Garcinia biflavonoids (for GB1, R is H; GB2, R is OH) GB1 (3,4,4,5, 5,7,7-heptahydroxy-3,8-biflavanone) and GB2 (3,4,4,5, 5,5,7,7-hexahydroxy-3,8-biflavanone).

*Garcinia kola*, a medium-sized tree found in most forests, belongs to the family* Guittiferae* and is commonly referred to as bitter kola.* G. kola *has been popularized as a “wonder plant” among South Western Nigerians because every part of it has been found to be of some medicinal value [4].* G. kola* is commonly used in folklore remedies for the treatment of ailments such as liver disorders, hepatitis, diarrhoea, laryngitis, bronchitis, and gonorrhoea [5]. It contains a complex mixture of biflavonoids, prenylated benzophenones, and xanthones [6]. Garcinia biflavanones (GB1 and GB2) (Figure 1) and kola flavanones are the major components of* G. kola* [7]. The therapeutic potential of* G. kola* seeds has been widely reported, including hepatoprotective effects [8] and treatment of cirrhosis and hepatitis [9, 10]. Even though the seeds and leaves of the plant enjoy considerable importance in folk medicine, to our knowledge, there is scant scientific information on the leaf of the plant compared to the seed, despite being called “wonder plant.” In our continuing efforts to isolate a potential natural proactive agent for the treatment of oxidative related diseases without attendant toxicity, this study, however, sought to evaluate the* in vitro* antioxidant potential of the MLE of the leaf of the plant and its* in vivo* effects in male albino rats.

## 2. Materials and Methods

### 2.1. Chemicals

Trichloroacetic acid (TCA), ferrous sulphate (FeSO_4_), thiobarbituric acid (TBA), sodium dodecyl sulfate (SDS), Folin-Ciocalteu reagent, glacial acetic acid, potassium ferricyanide, quercetin, gallic acid, ascorbic acid, azinobis-3-ethyl-benzothiazoline-6-sulfonic acid (ABTS^+^), and 1,1-diphenyl-2-picrylhydrazyl (DPPH) were procured from Sigma Co. (St. Louis, MO) USA. All other reagents used were of analytical grade.

### 2.2. Experimental Animals

All experiments involving rats were performed in accordance with prescribed ethics of animal use and investigator obligations (Guide for the Care and Use of Laboratory Animals 8th edition, 2011, Institute for Laboratory Animal Research, National Research Council, Washington, DC: National Academy Press; http://www.nap.edu). Rats weighing between 180 and 200 g were used for this investigation. The animals were fed with rat chow pellets and water* ad libitum* and maintained under a 12 h light and 12 h dark photo cycle throughout the period of the experiment.

### 2.3. Plant Material

Fresh* G. kola *leaves were collected from the land surrounding the College of Health Sciences, LAUTECH, Ogbomosho, Oyo State, Nigeria, and were authenticated by Dr. A. T. J. Ogunkunle, Department of Pure and Applied Biology, of the same institution.

### 2.4. Extraction Procedure

The powdered leaves of* G. kola *(1 kg) were exhaustively extracted with 3000 mL of 70% methanol. Methanolic leaf extract (MLE) was concentrated using a rotary evaporator. The percentage yield of the extract was calculated as

(1) $$\begin{array}{c} \frac{\text{Dry}\;\text{weight}\;\text{of}\;\text{the}\;\text{extract}}{\text{Powdered}\;\text{leaves}\;\left(1\;\text{kg}\right)}\times 100. \end{array}$$

### 2.5. Experimental Protocol

Rats for this study were randomized into three groups of six rats each. Groups I, II, and III were given distilled water and extract 100 mg/kg and 200 mg/kg body weight, respectively, through oral gavage for 14 consecutive days. All the animals were sacrificed by cervical dislocation 24 hr after the last exposure to the MLE of* G. kola*. Blood was obtained through heart puncture and collected into heparinized tubes and the livers of the rats were also removed. A small portion of the liver was fixed in 10% formalin for histological examination. The remaining part of the liver was washed in 1.15% KCl and blotted dry. Rat liver homogenates were prepared in potassium phosphate buffer (10 mM, pH 7.4; 1 : 4 w/v) using a homogenizer fitted with a Teflon pestle. The homogenates were centrifuged at 9,000 g for 10 min to obtain the postmitochondrial fraction (PMF) which was used to evaluate reduced glutathione (GSH), superoxide dismutase (SOD), glutathione peroxidase (GPx), and lipid peroxidation.

### 2.6. *In Vitro* Antioxidant Assay

The hydroxyl radical scavenging capacity of the MLE was evaluated according to an established method of Halliwell and Grootveld [11]. Superoxide anion scavenging ability, DPPH and ABTS were determined according to the methods of Beauchamp and Fridovich [12], Mensor et al. [13] and Re et al. [14] respectively. Inhibition of lipid peroxidation in egg yolk, rat liver, and brain homogenates as described by Ruberto et al. [15] was followed whereas total phenol and flavonoids were estimated by the methods of McDonald et al. [16] and Zhishen et al. [17], respectively.

### 2.7. *In Vivo* Study

The manual methods described by Cheesbrough [18] were used to evaluate hematological constituents. The concentrations of plasma glucose, magnesium, urea, creatinine, *γ*GT, ALT, and AST were determined with kits from Chemelex, S.A. 08420 Canovelles, Barcelona (Spain). Activities of rat hepatic GSH, SOD, and GPx were determined as previously described [19], Misra and Fridovich [20] and Rotruck et al. [21], respectively, while lipid peroxidation was detected as thiobarbituric acid reactive species and calculated as malondialdehyde (MDA) as described earlier [22].

### 2.8. Statistical Analysis

Data are expressed as mean ± standard deviation (SD) from the 6 rats in each group. The results were analysed statistically using one-way analysis of variance (ANOVA) followed by Dunnett's test. The minimum level of significance was fixed at *P* < 0.05. The 50% inhibitory concentration (IC_50_) of MLEs was estimated from regression analysis of concentration-inhibition curves using GraphPad Prism version 5.02 statistical software (GraphPad Software, San Diego California USA, http://www.graphpad.com). In this study, the IC_50_ value is defined as the concentration of extract required to scavenge 50% free radicals and is inversely proportional to the activity of the extract.

## 3. Results

### 3.1. Percentage Extract Yield

The weight of the dried methanolic leaf extract obtained was 223.5 g. The percentage yield of the extract was 22.35%.

### 3.2. Hydroxyl Radical, Superoxide Anion, DPPH, and ABTS^•+^ Scavenging Activities

The MLE of* G. kola *showed inhibition of hydroxyl radical scavenging activity with an IC_50_ value of 748.0 *μ*g/mL compared with an IC_50_ value of 45.2 *μ*g/mL for quercetin (Table 1). The extract scavenged superoxide anion with an IC_50_ of 10.0 *μ*g/mL, compared to ascorbic acid IC_50_ of 2.3 *μ*g/mL (Table 2). The MLE of* G. kola* exhibited DPPH scavenging activity with IC_50_ value of 126.0 *μ*g/mL while ascorbic acid yielded an IC_50_ value of 5.4 *μ*g/mL (Table 3). ABTS^•+^ was inhibited by the extract in a dose-dependent manner with an IC_50_ value of 34.3 *μ*g/mL while ascorbic acid showed an IC_50_ value of 5.7 *μ*g/mL (Table 4).

### 3.3. Inhibition of* In Vitro* Lipid Peroxidation in Egg Yolk, Rat Liver, and Brain

Table 5 shows that the MLE of* G. kola *inhibited lipid peroxidation in the egg yolk with an IC_50_ value of 190.0 *μ*g/mL while the IC_50_ value for ascorbic acid was 52.2 *μ*g/mL. The extract inhibited lipid peroxidation induced by FeSO_4_ in rat liver homogenate in a concentration-dependent manner (Table 6). The IC_50_ value of inhibition of lipid peroxidation by the extract was 218.0 *μ*g/mL while ascorbic acid (the standard) produced an IC_50_ value of 40.6 *μ*g/mL. FeSO_4_-induced lipid peroxidation in rat brain homogenate was similarly inhibited by the extract in a dose-dependent manner as shown in Table 7 with an IC_50_ value of 228.0 *μ*g/mL while ascorbic acid showed IC_50_ value of 36.5 *μ*g/mL.

### 3.4. Total Phenol and Flavonoid Contents

Total flavonoid content of the MLE of* G. kola* expressed as *μ*g/mg quercetin equivalent was 0.902 while total phenol content expressed as *μ*g/mg gallic acid equivalent was 1.196.

### 3.5. Hematological Analysis

Hematological parameters are shown in Table 8. The hematological results show that the extract did not induce significant changes in the hematological parameters evaluated. The extract at 100 mg/kg induced increase in the white blood cell count (WBC) and reduction was observed in hemoglobin (Hb). These changes were, however, not significant when compared with other groups.

### 3.6. Biochemical Analysis

Biochemical parameters are presented in Table 9. The plant extract did not have significant effects on some of biochemical parameters evaluated. However, insignificant (*P* < 0.05) decreases in plasma creatinine and magnesium occurred while a significant (*P* < 0.01) decrease in plasma glucose at 200 mg/kg extract was observed.

### 3.7. Effect of the Extract on Rat Hepatic GSH Levels

**Figure 2:**
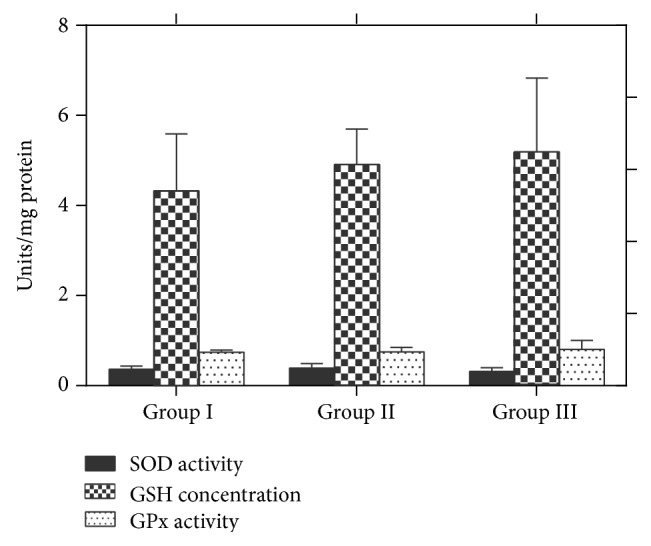
Hepatic GSH concentration and SOD and GPx activities in liver homogenates prepared from rats treated with distilled water as control (Group I), 100 mg/kg (Group II) or 200 mg/kg (Group III) MLE of* G. kola*. Data are presented as means ± SD (*n* = 6).

Administration of the extract to rats caused an increase in GSH levels compared to the control group (Figure 2). The percentage increase in GSH concentration of 100 mg/kg and 200 mg/kg extract was 13.7% and 20.1%, respectively (Figure 2).

### 3.8. Effect of the Extract on Rat Hepatic SOD and GPx Activities and Lipid Peroxidation

**Figure 3:**
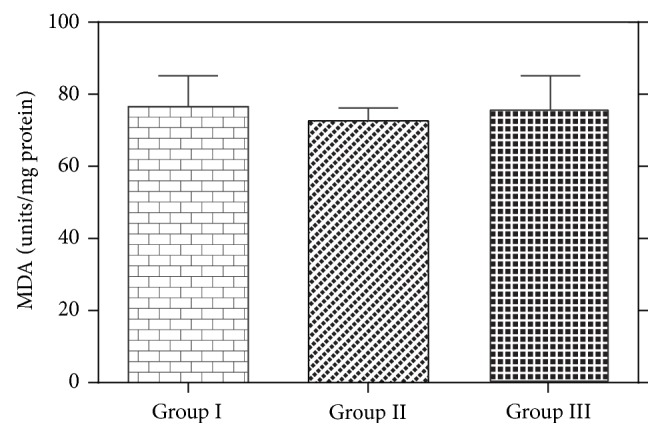
Hepatic lipid peroxidation measured as malondialdehyde (MDA) generated in liver homogenates prepared from rats treated with distilled water as control (Group I), 100 mg/kg (Group II) or 200 mg/kg (Group III) MLE of* G. kola*. Data are presented as means ± SD (*n* = 6).

**Figure 4:**
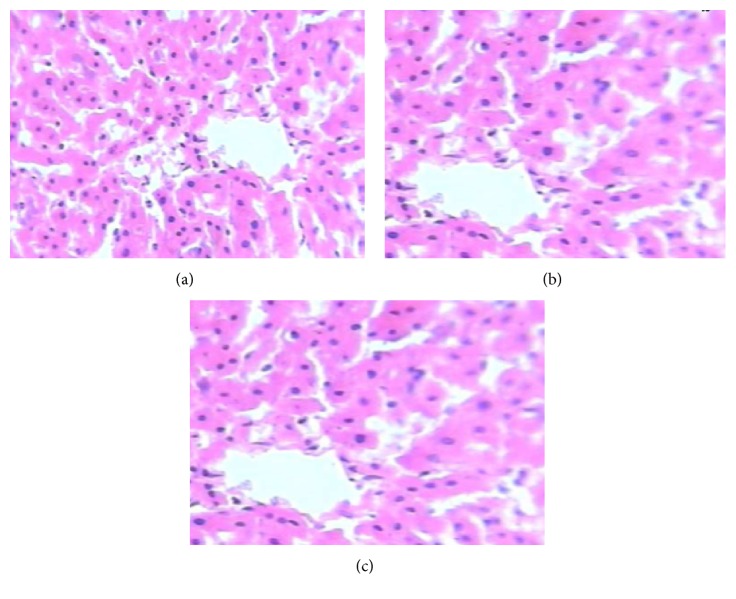
Histopathology of livers from rats in each treatment group. (a) Group I, distilled water control; (b) Group II, 100 mg/kg; and (c) Group III, 200 mg/kg MLE of* G. kola*. A portion of liver was fixed in 10% formalin and embedded in paraffin. Each liver section of 4-*μ*m thickness was stained with hematoxylin and eosin for light microscopic assessment at 100x.

The extract at both doses (100 mg/kg and 200 mg/kg) did not induce significant changes in hepatic SOD activities compared with the control group. However, the extract at 100 and 200 mg/kg showed significant (*P* < 0.05) increases in the activity of GPx by 1.08% and 7.83%, respectively, compared with the control group (Figure 2). The extract did not cause any increase in thiobarbituric reactive oxygen species as measured by malondialdehyde, a marker of lipid peroxidation (Figure 3). The results of histopathological analysis of liver sections prepared from rats in each treatment group are depicted in Figure 4. The MLE did not adversely affect the liver tissues as evidenced by well-preserved hepatocytes, nonvacuolated cytoplasm, well-demarcated sinusoids, no area of necrosis, and no fatty degeneration.

## 4. Discussion

Hydroxyl radicals are known to damage cell membrane through initiation of peroxidation of its lipid components [23]. Hydroxyl radicals are produced by the reaction of ferric (Fe^3+^)-EDTA, H_2_O_2_, ascorbic acid, and deoxyribose. The extent of inhibition of deoxyribose degradation in the presence of an agent gives an indication of hydroxyl radical scavenging ability. The MLE of* G. kola *exhibited dose-dependent inhibition of hydroxyl radical formation (Table 1). This result suggests that the extract has the potential to circumvent hydroxyl radical induced initiation of membrane lipid peroxidation.

Superoxide anion is a relatively weak oxidant generated in aerobic cells with the ability to transform into more reactive species such as H_2_O_2_, OH^•^, and singlet oxygen, which induce oxidative damage in lipids, proteins, and DNA [24], and has been implicated in several pathophysiological processes [25]. The riboflavin/NBT/illumination system generates superoxide anion which reduces the yellow dye (NBT^2+^) to a blue formazan product that can be measured spectrophotometrically at 560 nm. The superoxide anion scavenging property exhibited by* G. kola* may be due to neutralization of O_2_^−^ radical via hydrogen release and reduction of NBT by various phytochemical constituents in the extract. The inhibition of superoxide anion by the MLE of* G. kola* leaf may be of biological significance in terms of the cellular damage attributed to superoxide anion radical.

1,1-Diphenyl-2-picrylhydrazyl radical (DPPH) reacts in the presence of antioxidants to form 1,1-diphenyl-2-picryl hydrazine. The degree of discoloration from deep purple to yellow indicates the extent of radical scavenging potential of the antioxidant [26]. The MLE of* G. kola* and ascorbic acid exhibited DPPH radical scavenging activities in a concentration-dependent manner. The DPPH scavenging activity of the extract may be due to its ability to donate hydrogen ions to stabilize free radicals. Thus, the hydrogen donating property is central to the neutralization of free radicals that initiate oxidation processes and termination of radical chain reaction [27].

ABTS^•+^ radical reactions involve electron transfer and take place at a faster rate compared with DPPH radicals. ABTS^•+^ generation involves direct production of a blue/green chromophore through reaction between ABTS and potassium persulfate to give an absorbance of 0.750 at 734 nm in 0.1 M sodium potassium buffer (pH 7.4). The MLE of* G. kola* showed remarkable concentration-dependent inhibition of ABTS^•+^ which shows that the extract can participate in electron transfer, in addition to its H-donating property as found in DPPH radical scavenging.

Malondialdehyde is formed by oxidation of polyunsaturated fatty acids during the lipid peroxidation process and reacts with two molecules of thiobarbituric acid to produce a pinkish red chromogen [28]. This study evaluated the effects of the MLE of* G. kola *on egg yolk, rat liver and brain homogenates in the presence of FeSO_4_, a ROS-generating system. The extract showed significant inhibition of lipid peroxidation in all the tissue preparations tested. The inhibition of lipid peroxidation exhibited by the extract, though lower compared to the standard used, could be attributed to the presence of phytochemicals present in the leaves. This implies that the extract has potential to protect the lipid membrane constituents of liver, brain, and egg from Fe^3+^-induced lipid peroxidation.

The antioxidant properties of vegetables and fruits are significantly correlated with flavonoids, a class of secondary plant phenolics [24]. The flavonoids content of the methanolic extract of* G. kola* leaves in terms of quercetin equivalent was 0.90 ± 0.07 *μ*g/mg QE and total phenol was 1.20 ± 0.07 *μ*g/mg GAE. The antioxidant activities observed in this study may relate to the flavonoid and phenolic constituents in the leaf extract.

Hematological parameters are used to assess the effects of xenobiotics such as plant extracts on the well-being of animals [29]. Hematological profiles and biochemical indices are useful and reliable predictive indicators of toxicity in humans [30]. In this study, the plant extract did not show any significant effect on the blood RBC, WBC, PCV, Hb, MCHC, MCV, and MCH. However, it is remarkable to note that the extract at 100 mg/kg body weight induced increases in WBC and decreases with Hb more than what is observed at 200 mg/kg extract. Medicinal compounds or drugs have been shown to alter the normal range of hematological parameters either positively or negatively [31, 32].

Plasma AST, ALT, and GGT evaluated in this study show no significant difference in the treated groups compared to the control group. This shows that the MLE at both doses did not induce significant adverse effects on the integrity of hepatic cell membrane. AST, ALT, and GGT are enzymes that are released into circulating blood when the structural integrity of hepatic cells is compromised and therefore are used as biomarkers to evaluate hepatocellular insult [33]. There was a notable decrease in plasma glucose and magnesium in the groups treated with the extract at both concentrations. The decrease observed could be as a result of increased utilization of plasma glucose or enhanced mobilization of the glucose from the plasma to the tissues by the extract. The MLE at 200 mg/kg induced significant reduction in plasma creatinine while there was no significant effect observed in plasma urea. The observed decrease in creatinine in this study might have been mediated by flavonoids present in the extract. This implies that the leaves can enhance the functional capacity of the kidney.

GSH (*γ*-glutamyl-cysteinyl-glycine or glutathione), a water-soluble tripeptide containing a thiol group, is a potent reducing agent [34]. It has strong antioxidant properties that are essential in the detoxification of a variety of radicals. GSH is used as a cofactor by multiple peroxidase enzymes to detoxify peroxides generated from oxygen radical metabolism [35]. In the present study, the MLE of* G. kola* significantly (*P* < 0.05) increased hepatic GSH concentration by 13.7% and 20.1% at 100 and 200 mg/kg, respectively. Thus, the leaf extract may modulate glutathione metabolism via secondary plant metabolites (bioflavonoids and xanthones).

SOD catalyzes the dismutation of superoxide anion by converting it to hydrogen peroxide [36]. It is the primary catalytic cellular defence that protects cells and tissues against potentially destructive reactions of superoxide radicals and their derivatives. SOD can be induced when cells are exposed to agents that stimulate oxidative stress. The results obtained in this study show that oral administration of the MLE of* G. kola* did not have a significant effect on the hepatic SOD compared with the control group. This result further suggests that the extract did not cause any increase in superoxide anion that could induce the synthesis or degeneration of SOD.

Glutathione peroxidase (GPx) is a selenoenzyme responsible for the elimination of hydrogen peroxide and singlet oxygen [37]. Significant increases (*P* < 0.05) in GPx activities were observed in rats treated with the extract at 100 and 200 mg/kg body weight, which shows that the extract at both doses did not adversely affect the activity of GPx. Lipid peroxidation is a chain reaction initiated by free radical through the oxidation of polyunsaturated fatty acid. Malondialdehyde is an accumulated product of peroxidation of long chain fatty acids [38]. Lipid peroxidation in biological systems is one of the major mechanisms of cell injury in aerobic organisms subjected to oxidative stress [39]. Methanolic extracts of* G. kola *at both doses (100 and 200 mg/kg) lowered the formation of malondialdehyde in rat liver homogenates compared with the control. This result implies that the leaf extract at both doses maintains hepatic cell integrity.

Histopathological analysis of formalin fixed and paraffin-embedded liver sections shows that the extract at both concentrations (100 and 200 mg/kg) was well tolerated by the hepatic tissues of rats.

## 5. Conclusion

The* in vitro* and* in vivo* effects observed in this study might be linked to the reported phenolic compounds in MLE of* G. kola*. The mechanisms of the* in vitro* antioxidant and hypoglycaemic effects of the leaf extracts should be studied further in cell culture systems and animal models to enhance our understanding of the physiological significance of our findings and to clarify the usefulness of the* G*.* kola* leaves in traditional medicine practices.

## Figures and Tables

**Table 1:** Hydroxyl radical scavenging activity of the methanolic extract of *Garcinia kola *and quercetin.

Sample	Concentration (*µ*g/mL)	% Inhibition (*n* = 6)	IC_50_ (*µ*g/mL)	Regression equation
*Garcinia kola *	400.0	44.30 ± 0.01	748.0	*y* = 0.0144*x* + 39.21 (*r* ^2^ = 0.943)
	500.0	47.10 ± 0.08		
	600.0	48.10 ± 0.05		
	800.0	50.40 ± 0.04		

*Quercetin *	10.0	18.20 ± 0.10	45.2	*y* = 0.752*x* + 7.34 (*r* ^2^ = 0.806)
	20.0	23.00 ± 0.03		
	30.0	26.00 ± 0.40		
	40.0	30.00 ± 0.01		
	50.0	52.30 ± 0.02		

Results are expressed as means ± SD of five parallel measurements.

**Table 2:** Superoxide anion scavenging activity of the methanolic extract of *Garcinia kola* and ascorbic acid.

Sample	Concentration (*µ*g/mL)	% Inhibition (*n* = 6)	IC_50_ (*µ*g/mL)	Regression equation
*Garcinia kola *	3.30	26.50 ± 0.16	10.0	*y* = 3.30*x* + 17.1 (*r* ^2^ = 0.960)
	6.70	42.70 ± 0.12		
	10.00	50.50 ± 0.10		
	13.30	55.70 ± 0.02		
	16.70	75.10 ± 0.07		

*Ascorbic acid *	0.67	09.5 ± 0.12	2.3	*y* = 24.4*x* − 4.44 (*r* ^2^ = 0.954)
	1.33	24.8 ± 0.36		
	2.00	52.5 ± 0.22		
	2.67	63.9 ± 0.03		
	3.30	70.3 ± 0.01		

Results are expressed as means ± SD of five parallel measurements.

**Table 3:** DPPH scavenging activity of the methanolic extract of *Garcinia kola* and ascorbic acid.

Sample	Concentration (*µ*g/mL)	% Inhibition (*n* = 6)	IC_50_ (*µ*g/mL)	Regression equation
*Garcinia kola *	50.0	22.60 ± 0.03	126.0	*y* = 0.322*x* + 9.87 (*r* ^2^ = 0.972)
	150.0	64.30 ± 0.17		
	200.0	75.40 ± 0.80		
	250.0	86.40 ± 0.06		

*Ascorbic acid *	5.0	49.20 ± 0.12	5.4	*y* = 1.24*x* + 43.6 (*r* ^2^ = 0.939)
	10.0	53.90 ± 0.07		
	15.0	66.00 ± 0.03		
	20.0	69.40 ± 0.07		
	25.0	72.40 ± 0.02		

Results are expressed as means ± SD of five parallel measurements.

**Table 4:** ABTS^•+^ scavenging activity of the methanolic extract of *Garcinia kola* and ascorbic acid.

Sample	Concentration (*µ*g/mL)	% Inhibition (*n* = 6)	IC_50_ (*µ*g/mL)	Regression equation
*Garcinia kola *	7.50	28.60 ± 0.08	34.3	*y* = 0.653*x* + 27.7 (*r* ^2^ = 0.929)
	15.00	41.30 ± 0.07		
	30.00	50.60 ± 0.05		
	45.00	52.90 ± 0.07		
	60.00	67.90 ± 0.04		

*Ascorbic acid *	1.50	25.20 ± 0.08	5.7	*y* = 6.335*x* + 14.1 (*r* ^2^ = 0.970)
	3.75	34.10 ± 0.07		
	6.00	55.00 ± 0.06		
	7.50	60.90 ± 0.08		

Results are expressed as means ± SD of five parallel measurements.

**Table 5:** Inhibition of lipid peroxidation in egg yolk homogenate by methanolic leaf extract of *Garcinia kola* and ascorbic acid.

Sample	Concentration (*µ*g/mL)	% Inhibition (*n* = 6)	IC_50_ (*µ*g/mL)	Regression equation
*Garcinia kola *	94.24	32.90 ± 0.03	190.0	*y* = 0.149*x* + 21.8 (*r* ^2^ = 0.896)
	142.86	47.10 ± 0.02		
	190.48	51.10 ± 0.01		
	238.10	55.30 ± 0.02		

*Ascorbic acid *	09.50	20.60 ± 0.02	52.2	*y* = 0.732*x* + 11.9 (*r* ^2^ = 0.922)
	19.00	26.10 ± 0.03		
	28.57	31.10 ± 0.04		
	38.00	35.40 ± 0.04		
	47.62	50.80 ± 0.01		

Results are expressed as means ± SD of five parallel measurements.

**Table 6:** Inhibition of lipid peroxidation in rat liver homogenate by methanolic leaf extract of *Garcinia kola* and ascorbic acid.

Sample	Concentration (*µ*g/mL)	% Inhibition (*n* = 6)	IC_50_ (*µ*g/mL)	Regression equation
*Garcinia kola *	94.24	24.00 ± 0.02	218.0	*y* = 0.200*x* + 6.64 (*r* ^2^ = 0.961)
	142.86	36.00 ± 0.03		
	190.48	48.00 ± 0.04		
	238.10	52.00 ± 0.06		

*Ascorbic acid *	9.50	5.40 ± 0.02	40.6	*y* = 1.62*x* − 15.4 (*r* ^2^ = 0.912)
	19.00	10.60 ± 0.11		
	28.57	32.58 ± 0.40		
	38.00	35.80 ± 0.05		
	47.62	70.00 ± 0.09		

Results are expressed as means ± SD of five parallel measurements.

**Table 7:** Inhibition of lipid peroxidation in rat brain homogenate by methanolic leaf extract of *Garcinia kola* and ascorbic acid.

Sample	Concentration (*µ*g/mL)	% Inhibition (*n* = 6)	IC_50_ (*µ*g/mL)	Regression equation
*Garcinia kola *	94.24	18.0 ± 0.01	228.0	*y* = 0.259*x* − 8.40 (*r* ^2^ = 0.985)
	142.86	26.0 ± 0.04		
	190.48	42.0 ± 0.01		
	238.10	54.0 ± 0.03		

*Ascorbic acid *	9.50	18.0 ± 0.02	36.5	*y* = 1.14*x* + 8.54 (*r* ^2^ = 0.959)
	19.00	28.0 ± 0.06		
	28.57	47.0 ± 0.03		
	38.00	53.0 ± 0.01		
	47.62	60.0 ± 0.05		

Results are expressed as means ± SD of five parallel measurements.

**Table 8:** Haematological indices of rat dosed with methanolic leaf extract of *Garcinia kola* at 100 and 200 mg/kg for 14 days.

Groups	RBC (×10^12^/L)	WBC (×10^9^/L)	PCV (L/L)	HB (g/L)	MCHC (%)	MCV (fl)	MCH (pg)
I	5.09 ± 0.31	4.78 ± 0.65	0.42 ± 0.05	170.20 ± 17.98	411.80 ± 53.80	81.94 ± 10.04	33.46 ± 3.07
II	5.27 ± 1.13	6.88 ± 2.65	0.39 ± 0.03	151.60 ± 17.69	393.60 ± 61.80	73.56 ± 10.74	30.04 ± 7.44
III	5.43 ± 0.82	5.45 ± 1.21	0.40 ± 0.04	165.75 ± 15.15	412.00 ± 22.00	75.58 ± 14.92	30.95 ± 4.43

Data are expressed as mean ± SD (standard deviation) of six animals in each group. RBC (red blood cell), WBC (white blood cell), PCV (packed cell volume), HB (hemoglobin), MCHC (mean corpuscular hemoglobin concentration), MCV (mean corpuscular volume), and MCH (mean corpuscular hemoglobin). (Group I), 100 mg/kg (Group II), or 200 mg/kg (Group III) MLE of *G. kola*.

**Table 9:** Biochemical indices of rat dosed with methanolic leaf extract of *Garcinia kola* at 100 and 200 mg/kg for 14 days.

Groups	UREA (mg/dL)	AST (IUL^−^)	ALT (IUL^−^)	*γ*-GT (IUL^−^)	Creatinine (mg/dL)	GLU (mg/dL)	MAG (mg/dL)
I	44.09 ± 6.57	27.80 ± 2.78	22.40 ± 2.19	4.00 ± 1.21	3.09 ± 0.85	40.78 ± 5.60	2.11 ± 0.35
II	45.13 ± 3.28	28.00 ± 3.08	23.20 ± 9.01	4.17 ± 1.02	2.46 ± 1.16	36.09 ± 2.90	1.73 ± 0.12
III	42.49 ± 3.76	27.20 ± 4.09	21.20 ± 5.22	5.06 ± 1.31	1.60 ± 0.77	30.85 ± 8.60	1.60 ± 0.02

Data are expressed as mean ± SD (standard deviation) of six animals in each group. AST (aspartate transaminase), ALT (alanine transaminase), *γ*-GT (gamma glutamyl transpeptidase), GLU (glucose), and MAG (magnesium). (Group I), 100 mg/kg (Group II), or 200 mg/kg (Group III) MLE of *G. kola*.
